# Supporting Vulnerable Populations During the Pandemic: Stakeholders’ Experiences and Perceptions of Social Prescribing in Scotland During Covid-19

**DOI:** 10.1177/10497323211064229

**Published:** 2021-12-30

**Authors:** Dr Alison Fixsen, Dr Simon Barrett, Michal Shimonovich

**Affiliations:** 1 164128 University of Westminster, London, UK; 2 5994 Newcastle University, Population Health Sciences Institute, Newcastle upon Tyne, UK; 3 3526 University of Glasgow, MRC/CSO Social and Public Health Sciences Unit, Glasgow, UK

**Keywords:** social prescribing, qualitative studies, Covid-19, third sector, vulnerable communities

## Abstract

Social prescribing schemes refer people toward personalized health/wellbeing interventions in local communities. Since schemes hold different representations of social prescribing, responses to the pandemic crisis will vary. Intersectionality states that social divisions build on one another, sustaining unequal health outcomes. We conducted and inductively analysed interviews with twenty-three professional and volunteer stakeholders across three social prescribing schemes in urban and rural Scotland at the start and end of year one of the pandemic. Concerns included identifying and digitally supporting disadvantaged and vulnerable individuals and reduced capacity statutory and third-sector services, obliging link workers to assume new practical and psychological responsibilities. Social prescribing services in Scotland, we argue, represent a collage of practices superimposed on a struggling healthcare system. Those in need of such services are unlikely to break through disadvantage whilst situated within a social texture wherein inequalities of education, health and environmental arrangements broadly intersect with one another.

## Introduction

Social prescribing is a salutogenic approach^1^ which allows healthcare and other professionals to refer patients toward health and wellbeing interventions and activities in the local community, usually with the assistance of a designated social prescribing coordinator (SPC) or ‘Links Worker’. Social prescribing approaches have been adopted in countries including the US, Canada, Finland and Brazil (Younan et al., 2020), but principally in the UK. Studies of health and social care carried out during Covid-19 have confirmed the unprecedented stresses on lay persons and health practitioners at this time (Bröer et al., 2021; Kamin et al., 2021; Marshall et al., 2021; Pierce et al., 2020). The pandemic has particularly affected people on low incomes and/or with poor health (Nicola et al., 2020; Pierce et al., 2020), who are generally those for whom social prescribing is intended. Since different social prescribing schemes hold different representations of social prescribing, their response to the Covid-19 pandemic and means of supporting disadvantaged and vulnerable populations at this time will vary. Before Covid-19, social prescribing services were almost exclusively in-person; however, lockdown rules and social distancing made this largely impossible. Scotland is the country with the highest level of deprivation in Europe (Morton, 2021) and recent data suggests health inequalities there to have only marginally reduced in the last decade (National Records of Scotland, 2020). A small body of literature has now focused on the work of health professionals during the pandemic (Kamin et al., 2021). How different professional and volunteer stakeholders in social prescribing schemes in urban and rural Scotland responded and adapted to the conditions of Covid-19) in a time of limited access to health resources has yet to be considered from a social constructionist perspective: this is the focus of our study.

The social constructionist approach takes a broad perspective of a dynamic society, regarding both perceived reality and knowledge as relative and as produced by groups and individuals making claims to phenomena at different times and in various locations (Berger & Luckman, 1966; Gergen, 1994; Burr, 2015). According to Gheradi (2012), knowledge does not just reside in the heads different people, it is anchored in the material world, through webs of social practice. Another analytical theory used to explain power and resource inequalities is that of intersectionality which, although originally focused on intersecting race and gender equalities (Cooper, 2016), has been extended to explain how multiple social divisions in a given society, including race, ethnicity, class, gender, sexuality, disability and age, build on and work together to sustain unequal health outcomes (Kapilashrami & Hankivsky, 2018). Together, these theories suggest that social prescribing is best viewed as a complex and shifting relational field in which different players, including politicians, policy makers, general practitioners (GPs), researchers, SPCs, community workers and patients, each make knowledge claims, but hold unequal power and privilege (Fixsen et al., 2020). The social and material world has recently undergone a rapid change; understanding its variable impact on individuals and groups in different geographical and social settings is crucial to future policies. Finally, by gathering our data from both urban and rural areas, we can add to comparative studies of social prescribing in different settings (Younan et al., 2020). We begin with our study background, including the aims and approaches of social prescribing, and social prescribing in Scotland under Covid-19 conditions.

### Social Prescribing and the Social Prescriber Coordinator

In its emphasis on collaborative, community-based healthcare, social prescribing appears to fit with a social constructionist interpretation of health and wellbeing (Polley, 2018; Fixsen et al., 2020). In other ways, social prescribing represents an eclectic assemblage – or collage – of models and services, usually based on ideas about personal empowerment supported through maximizing health promoting assets within community settings (Public Health England, 2019; Younan et al., 2020). The UK Department of Health introduced the term ‘social prescribing’ in 2006 to promote independence and health in community settings (Department of Health, 2006). It has since featured prominently in the NHS (National Health Service) ‘New Personalized Care Model’ (Health Improvement Scotland, 2019; NHS England, 2019). Early versions of social prescribing, on the other hand, were bottom up initiatives planned and delivered by neighbourhood community and voluntary groups linked with one or two local GPs outside of any government model. Variations in the terminology have also caused some confusion; social prescribing has been used interchangeably with ‘community referral’ and ‘linking scheme’ (Hassan et al., 2020).

The emphasized goals of social prescribing vary with scheme and author but include; addressing non-medical concerns in specific populations (Baker & Irving, 2016), strengthening primary-care/third-sector partnerships (Brandling & House, 2009), lessening GP burden and mitigating health inequalities (Mercer et al., 2017). Recent schemes often utilize established health models, such as the Theory of Change model (SPRING, 2021) and the Model of Health and Wellbeing (Alliance for Healthier Communities, 2021) to guide service delivery. Despite these ambiguities, as a means of using community initiatives to promote health and wellbeing, social prescribing has continued to grow in popularity and across the globe.

### Key Stakeholders in Social Prescribing

Generally social prescribing includes four key stakeholders: patient, referrer, social prescriber coordinator (SPC) and community provider. The SPC (who is a central player in the NHS model) acts as the link person (SPCs working in NHS settings are referred to as Links Workers), working co-creatively with clients to connect them appropriately with community-based (third sector) interventions such as art, gardening or exercise classes or support organizations such as mental health, housing and welfare (Brandling & House, 2009; White et al., 2017; Fixsen & Polley, 2020). SPCs may be practice-attached (i.e. based in a general practice (GP) surgery and usually government funded), third-sector–attached (i.e. based in the community and usually funded by charities or private organizations) or a combination of both. SPCs usually receive referrals from GPs, pharmacists and health or social care professionals, although in some schemes people can self-refer (Bell et al., 2007; Husk et al., 2016; Mercer et al., 2019).

SPCs work with vulnerable individuals with complex social needs, frequently in poorer communities (Duffin, 2016; Mercer et al., 2019; Fixsen et al., 2020). They require a good local knowledge, person-centred skills and an understanding of boundary keeping (Frostick & Bertotti, 2019; Polley, 2018). Multiple studies have described the local successes of social prescribing initiatives (e.g. Stickley & Hui, 2012; Wildman et al., 2019); however, evaluations of social prescribing have been less positive about evidence concerning its overall outcomes (Bickerdike et al., 2017; Mercer et al., 2019; Public Health England, 2019). Unsurprisingly, the heterogeneity of populations and programs involved in social prescribing has made ascertaining which model of social prescribing works best and how particular interventions impact on recipients’ health and wellbeing difficult (Husk et al., 2016; Younan et al., 2020). Qualitative studies (including our own) have also indicated problems at local operational level, with individual practitioners struggling to put joined-up, collaborative working into practice due to inadequate feedback or trust issues (White et al., 2017; Fixsen et al., 2020). A tension also exists between the ethos of self-care and self-responsibility, which government-linked institutions in particular have attached to social prescribing in their promotion literature, and the ability of schemes to address health and social determinants related to inequality issues, such poor housing and unemployment in the absence of real structural change (Carlisle, 2010; Mackenzie et al., 2020). Despite the rhetoric around social prescribing (Bickerdike et al., 2017), further studies are required before its long-term benefits at society level can be assessed.

### Scotland and Covid-19

The Scottish Government has made a commitment to deliver 250 Links Workers over the life of its Parliament to primary-care/GP practices under the new GP (general practitioner) contract, across the country (Public Health Scotland, 2020). Glasgow is considered the most deprived city in Scotland; almost half of its residents live in the 20% most deprived areas in the country (Understanding Glasgow, 2021). The pandemic has further highlighted and exacerbated health inequalities within the country (Scottish Government, 2021), perpetuating the Inverse Care Law, which states that good medical care tends to vary inversely with need in the population served (Marmot, 2018). Social and economic effects of the Covid-19 pandemic have been shown to disproportionately affect low-skilled workers, low-income households, regardless of whether or not they contract the virus (Marshall et al., 2021). This in turn has had knock on effects, such as increased hardship, poorer mental and physical health, and record levels of drug related deaths in Scotland in the last year (BBC, 2021).

Health and social inequality issues also affect rural and isolated populations in Scotland. Yet, while there have been multiple studies of social prescribing in urban Scotland, especially Glasgow (e.g. Mercer et al., 2017; Mercer et al., 2019; Skivington et al., 2018; Mackenzie et al., 2020), social prescribing in rural and remote Scotland has only recently received academic attention.^2^ Recent census data suggest that the Western Isles has the greatest proportion of lone pensioner households in Scotland which, even without social isolation or shielding, presents a challenging situation (Western Isles Integration Joint Board, 2020). Studies suggest that living alone, as under social distancing measures related to Covid-19, can lead to decreased health and wellbeing (Kamin et al., 2021) and increased risk of dementia, depression and premature mortality in the elderly (Baker & Irving, 2016). It was for this reason that our study looked at both urban and rural social prescribing schemes.

### Digitalization and the Third Sector

Accelerated by the recent pandemic, interactive technology (IT) has become an essential part of the delivery of health and social services and this has multiple implications for deliverers and recipients of social prescribing (Fixsen et al., 2021). Social connection during lockdown is particularly difficult for people living in vulnerable circumstances, who may also experience digital exclusion. With this in mind, initiatives such as *Connecting Scotland* has been distributing iPads to the most vulnerable through GP surgeries, care homes and charities (Connecting Scotland, 2021). GP surgeries and social prescribing schemes have also been busy training their staff in IT skills and data protection measures (Sankeram, 2021). Even so, delivering social prescribing remotely is very new, with the full implications of this yet to be explored.

A further issue for social prescribing under Covid-19 is its reliance on a third sector which has also needed to rapidly digitalize. Funding cuts to local organizations were already flagged up as a problem in social prescribing studies before the pandemic (Mackenzie et al., 2020; Polley et al., 2019), with funding sources projected to decline across the third sector in 2021 (Delahunty, 2021). We turn now to our study, which is guided by the following question: How did different professional and volunteer stakeholders in social prescribing schemes in urban and rural Scotland respond and adapt to the conditions of Covid-19 in a time of limited access to health and community resources? And, how can a comparative study such as this inform us about intersectionality and health disadvantage in society?

## Methodology

### Study Context

The context of our study was social prescribing in Scotland, and more specifically Glasgow and the Western Isles during the first year of the Covid-19 pandemic. We captured responses from within three social prescribing schemes: The Community Links Worker (CLW) Program, SPRING social prescribing and, the mPower Community Navigator program. The CLW program, in Glasgow presently run through the Health and Social Care Alliance, supports the so-called Deep End general (primary care) practices – those located in the most socioeconomically deprived areas of the city (Mercer et al., 2019). Across Scotland there are over 100 designated Deep End practices, of which about 31 now have a practice-attached ‘Links Worker’ (Alliance Scotland, 2021).

SPRING social prescribing is a national partnership between Scottish Communities for Health and Wellbeing and the Healthy Living Centre Alliance in Northern Ireland, funded by the National Lottery. Our study focused on the Glasgow branches of the CLW and SPRING schemes. The Community Navigator scheme, managed through mPower, is a 5-year project supported by the European Union (mPower, 2020). It serves rural communities in Scotland and Ireland and focuses on two areas: social prescribing and digital literacy. Our study looked at the work of the Community Navigators (CNs) in the Western Isles of Scotland. Details of the programs/schemes are described in Supplementary Table 1. The different terminology used for social prescribing coordinators (SPCs) should be noted. Going forward, we will refer to SPCs associated with the CLW program as *Links Workers*, those associated with SPRING social prescribing as *SPRING advisors* and those associated with mPower as *CNs* (Community Navigators). When discussing the role in general or across the three schemes, we will continue to refer to them as SPCs.

### Data Collection

A purposive approach, with a mixture of convenience and snowball sampling, was used to recruit participants who had different interests and involvements in social prescribing, first in the Glasgow area and later the Western Isles of Scotland. A loosely structured interview guide (see Supplementary file interview protocol) was designed for the interviews, based on a similar guide used by Alison Fixsen in a previous study, but adapted to the conditions and background of participants. Due to the onset of Covid-19, additional questions concerning participants’ professional views and experiences of social prescribing under Covid-9 and post-Covid were also added. All twenty-three participants were first contacted by email by the first author, who explained the researchers’ backgrounds and interests in the study and attached a Participant Information and Consent Form. Due to social distancing conditions, all interviews were conducted individually by the first author via Skype or telephone. Digital interviewing lacks certain qualities of face-to-face interviewing such as a controlled environment. Digital interviews during the pandemic excluded travel problems and physical safety risks, and in our case were much more convenient for home-working participants. All those contacted agreed to be interviewed. Originally, fieldwork was also to include in-person focus group interviews with service users; however, due to the lockdown, National Health Service (NHS) data was unavailable and professional interviews were carried out remotely. While we recognize the limitations of this in terms of a wider perspective, it allowed us to focus more on the operators within different schemes.

Between March and mid-June 2020, Alison conducted twenty-two semi-structured interviews with a range of professional stakeholders in social prescribing, first in Glasgow and later in the Western Isles of Scotland. Participants in round one included SPCs from the three different schemes, GPs, social prescribing managers, researchers and representatives of third-sector organizations. After the initial interviews and coding, a gap of 7 months occurred due to a period of illness. Given the continued disruption to life in the UK during this period, it was considered expedient to conduct follow-up interviews with a sample of participants selected on the basis of availability and representativeness to a scheme or group. In all, seven participants were re-interviewed in January/March 2021, to find out what had changed in terms of their work arrangements including channels of communication, reasons for referral and relationships with the third sector. In addition, one new interview was conducted with an SPC who worked extensively with clients whose first language was not English (see Supplementary Table 2*: participants)*, All interviews were one-to-one, semi-structured and conducted remotely by the project lead on a secure audio-visual platform or telephone. Interviews ranged from 40 minutes to an hour. Collection of data was carried out until a satisfactory level of saturation had been reached (i.e. it was felt that coding and themes were sufficient). Participants were given the opportunity to review their transcript if they chose. Transcripts were professionally transcribed.

*Ethics:* All parts of the study were approved by the University of Westminster Liberal Arts and Sciences Ethics Committee. All participants were supplied with participant information sheets and gave their consent to the interviews being recorded and for extracts of interview data to be used. All data use adheres strictly to the terms of the Data Protection Act (DPA 2018). Only pseudonyms are used in this study. Due to the specialist nature of professional roles, the Ethics Committee suggested that data such as age and place of residence not be mentioned in published materials. However, given the limited number of people in post, participants were made aware before interviews that their anonymity might be partial rather than total.

### Analysis

**Figure 1.**
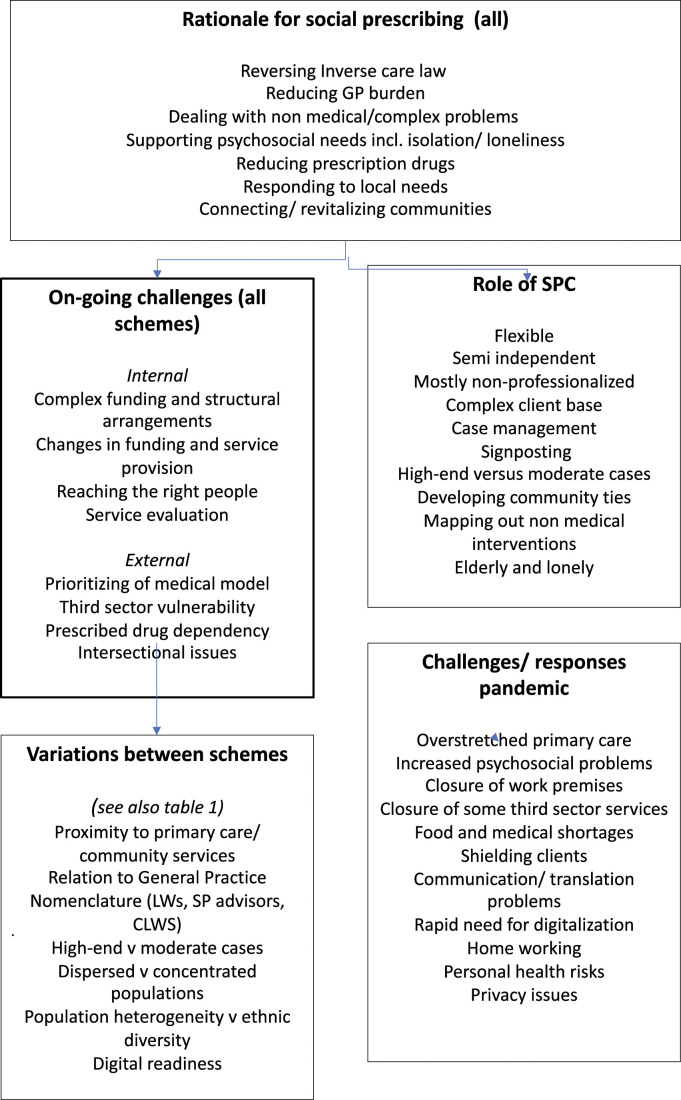
Main themes from findings.

All three authors contributed to the analytic process, along with the identification of literature sources and the conceptualization of ideas discussed in the paper. An inductive thematic analysis was used to examine the initial set of data, and to subsequently amend and add to it based on the follow-up interviews. Braun and Clarke (2006) suggest that while thematic analysis is often described as a realist, experiential method, it is also compatible with a constructionist position. Consistent with the constructionist approach, the language and metaphors used by participants to convey ideas were emphasized. We also used aspects of grounded theory, such as beginning the study without any recognized theoretical framework and using theoretical sampling in the selection of participants (Strauss & Corbin, 2015). Simon Barrett worked on ‘creating order’ (Spencer et al., 2003) within the large dataset, transcripts were thoroughly coded and categorized into themes. In practice, this involved the Alison listening to the audio-recordings multiple times and making memos, then each member of the team reading and re-reading transcripts to familiarize ourselves with the data, making notes of recurrent themes within and across participants’ transcripts. Themes were then discussed between the team. Each transcript was also uploaded to NVivo 12 by the third author to ensure a systematic approach to the analysis, and to extract further codes and themes. Figure 1 illustrates the main themes from our findings.

Data from each participant was mapped out under corresponding themes, by way of a framework matrix. This process allows the authors to assess the distribution of data across the sample and identify commonalities or disparities in responses, as well as the identification of gaps in the data which could inform the second round of interviews. As further themes emerged from each transcript, new codes were created accordingly, in an iterative process. It was then possible to start to develop explanations from the data and explore its implications.

### Findings

#### The Three Schemes

Situated in areas of concentrated deprivation, the Links Worker scheme had originally been conceived of both as a way of reversing the Inverse Care Law and reducing the burden on overstretched GPs in these areas. Prior to the scheme, one GP explained how, ‘We’ (the practice GPs) ‘all tried to basically do social prescribing’ in terms of referring patients to activities. To free up GP time, it had been decided early on in the program that any patient assigned to the surgery could be referred to the Links Worker, irrespective of age or condition, for example, ‘So, we’ve got about 5,000 patients and I see anybody from cradle to grave’.

GPs we interviewed regarded their Links Worker as valuable in supporting them and their patients, in particular those who came with psychosocial needs or who you ‘didn’t know what to do with’. However, ‘buy-in’ to social prescribing from GPs in general remained patchy – according to this Deep End GP; ‘We have probably been operating on a coalition of the willing’. Looked at from the outside, the rather open-ended role of the SPCs could also lead them into areas that some might consider to be beyond their role description, as this researcher explained:

We found in some instances…they [the Links Workers] were providing more of a case management role and being more involved in actually providing support rather than just linking people to services. *Researcher*

SPRING social prescribing advisors were also tasked with supporting GPs in their area; however, rather than working in GP practices, they operated in community settings, close to other activities run by the host organization. Unlike Links Workers, SPRING advisors did not deal with ‘high end’ (severe) medical or complex social cases. Instead, they based their work on the Theory of Change model (SPRING, 2021) and ‘cutting out’ the medical association, which, according to one advisor, clients could find ‘threatening’. Stakeholders in the Community Navigator scheme emphasized the use of personalized wellbeing plans and digital health interventions to help people with long-term conditions manage their own health and connect to them others in these remote communities; “And it’s really to help the people who may be in danger of being isolated or who are lonely, who are going to GPs, for example, on a fairly regular basis” (*manager).*

The emphasis on developing ties with the community was common to all schemes, both as a means of supporting patients and of strengthening the community itself. For example, a recognized function of the practice-attached Links Worker is ‘mapping out’ potential non-medical interventions in the community and thus acting as a repository of information for general practice staff (Mercer et al., 2019). In one GP surgery, this linking of the medical and community was encouraged through inviting different community project workers into the practice to make themselves known to staff and patients, serving the dual purpose of educating staff and attracting potential service users. Various obstacles, however, impeded the development of strong and lasting relationships between the medical and the third sector. In all three schemes, there were uncertainties about future funding of both social prescribing schemes and third-sector organizations. As one Links Worker explained, ‘We really don’t know what that’s going to look like ahead, unfortunately, we just don’t. And that’s a huge, huge worry’. Getting funding bodies to acknowledge the importance of community initiatives was challenging, as other areas of primary care were prioritized, with the community health sector usually afforded lesser importance than medical services. One manager’s experience had been that people and issues within the community sector tended to be treated as less important than those in primary care by policy makers and in planning meetings. As this SPRING advisor saw it:

It [requires] a culture shift…moving from the medical model of health to the social model of health…. it’s also trying [to] influence key players and strategic players who you know hold budgets to shift this model so that its community-led health. So that funding goes directly to these community-led health organizations to run social prescribing.

More high level, constructionist views about social prescribing were also expressed, principally by researchers and some GPs, concerning what some saw as a fundamental disconnect between the structural causes of inequality and the beliefs held by practitioners that, in individual ways, social prescribing was making a difference. As one researcher expressed it, ‘We know that the only real way we can address inequalities is upstream policies rather than just individualized [health plans/solutions]’.

### Operating in a Challenging Landscape

Even before Covid-19, social prescribing services in Glasgow faced major obstacles in tackling health inequalities. The Deep End GPs practices featured in this study were based in ‘the most socially, economically deprived communities in Glasgow’. Some GP practices served a large number of asylum seekers, who, as newcomers to Glasgow, frequently sought practical help from the Links Workers; ‘even just basic support such as setting up a bank account or places to go for children’. Routine cases for GPs and Links Workers included generations of families who suffered from multiple intersecting disadvantages, as this Links Worker explained:

We are working with loads of people that have maybe got substance abuse problems- because we are based in the communities where that is more prevalent that will be more prominent…And also ethnic minorities and marginalized groups. There’s a growing Roma community up in [area name removed]- we’re based in three practices there and they’re a very marginalized group.

Encouraging patients with multiple problems to come off their medication and consider alternatives such as exercise was fraught with difficulties, as this GP explained:

Our patients are so conditioned that they think sometimes medications will fix their problems, but we create more problems like what we know from the States… [we] know from the figures in the UK as well that painkillers are much more often prescribed in deprived areas and we see our patients being dependent on opioids.

The physical environment in which social prescribing schemes operated presented its own challenges and barriers. As one researcher commented, alongside social prescribing, ‘you need the regeneration of areas and you need the other support systems in place’. Participants spoke of gaps in local (council-run and voluntary) services, with some areas of funding ‘hugely cut up here in Glasgow’. Also, funding cycles could change rapidly, which could be unsettling and disheartening: ‘you apply for funding, it lasts two years’. At the same time, successful schemes serving clients of social prescribers, such as a popular park run scheme, had, according to one volunteer become a source of pride and by attracting international participants had helped to regenerate a struggling community.

Located in remote rural areas, the CNs working in the Western Isles faced their own challenges in terms of work isolation, travel distances and inclement weather conditions. Typically, CNs worked away from GP surgeries and other medical centres and travelling to far-away destinations meant they needed to have their own cars. Many of their clients were elderly and alone; thus, time on visits tended to be long. A round trip for one CN could last a day and in winter especially, travelling conditions could be difficult:

I did cancel visits the day that we had quite a bit of snow because of just the safety aspects. The roads here, some of them are so tight and windy and they don’t always get gritted if the conditions are going to be poor.

### Effects of the Pandemic

#### Initial Challenges

Of all those whose reality has been altered by Covid-19, the medical profession has been one of the most immediately affected. The pandemic quickly saw acute health services stretched to their limit. GPs were finding remote working difficult, and it had taken a while for Links Workers to get digitally connected to primary care systems. All three schemes had busied themselves in offering practical, psychological and digital support to existing and shielding clients. These efforts were appreciated by GPs caught up with acute medical care:

He [the Links Worker] has been invaluable during this Covid pandemic, even though he needs to work from home. But despite that he’s been amazing at liaising with third sector organizations, and patients, and mobilizing resources to mitigate some of the effects of the pandemic around practical things like foods deliveries, prescriptions-but also emotional support… mental support. *GP*

The reality of the SPRING social prescribing advisors had also altered during the pandemic. There had been an initial drop in GP referrals to SPRING advisors, who instead had taken on a broad health education and social role in their local communities, as one SPRING advisor explained:

So, we did do some online activity…cooking and nutrition classes and…Exercise, so yes, and…around the summer last year we done some afternoon tea, which was just a selection of sandwiches and cakes and things to offer to the most vulnerable…And at Christmas time we done some pamper packs.

Many activities of SPCs had been put on hold during social distancing. For example, it was common for Links Workers and SPRING advisors in Glasgow to accompany vulnerable clients to community or support centres. As one manager who worked for a charity supporting victims of abuse explained; ‘We get a lot of referrals from the Links practitioners and they also support people to access the service, so they will physically bring them to their appointments’. Not only was this impossible during lockdown but one Links Worker later reported that the charity had (temporarily or permanently) closed its doors.

### Remote Working

The digitalization of healthcare and social systems is a key to the new reality of a Covid-19 society. Loss of face-to-face contact between practitioners and clients was missed by both GPs and SPCs. According to one GP, the Links Worker scheme had been ‘working well [but] we are missing the direct contact with our Links Worker because they now work remotely’. Prior to Covid-19, Link Workers and SPRING advisors used little internet technology for their work, and so had needed to figure out how to use technological platforms themselves before educating clients about them. CNs working for mPower, on the other hand, had been distributing digital tablets to clients in remote areas before Covid-19, so were ahead of other schemes in this respect.

By the second round of interviews, Links Workers were also distributing iPads, and all SPCs were making use of video platforms. Video calls presented their own challenges, with technology sometimes failing and home working considered less of a professional and private space than an office. In addition, not all clients possessed digital devices or knew how to use them, and SPCs were often tasked with instructing clients on how to use them.

By early 20201, some SPCs had arranged in-person interactions with their clients, largely through Walk and Talk sessions in a local area. In most (but not all) areas of the Western Isles, CNs had been able to continue with face-to-face contact for initial meetings after the initial lockdown, with follow-ups face-to-face. According to one CN, social isolation had been a big issue for clients, and she had been offering ‘mild psychological support’ such as CBT (cognitive behavioural therapy), although the main focus had been digital assistance for people with limited skills. In her view, it was often things like connecting people to befriending services or to family that made the most difference to clients’ lives:

I’ve got one lady who self-referred in for digital support [with] absolutely no digital skills whatsoever, in her eighties, who would normally travel to see family in Australia…. but obviously can’t, and it was then getting her device set up and teaching her how to use that. Now she Facetimes her family regularly, actually she Facetimes me on a Saturday just for a wee chat.

Paradoxically, the enforced shift to online service provision and activities had benefitted those previously unable or reluctant to attend physical, in-person sessions. One Links Worker spoke of one lady who ‘doesn’t engage with anything in normal times because she doesn’t have the confidence…but I’ve linked her in with some online [yoga/Tai chi] sessions…and she’s really keen on that’. It seemed likely that some services would continue online, even post-pandemic.

### Increased Need/Diminished Resources

While having grown rather accustomed with what had become popularly known as the ‘new normal’, the first year of Covid-19 in Deep End practices GP had been ‘very, very intense’, and had been especially challenging where there were language barriers. Before Covid-19, there would be a live language interpreter in GP and Links Worker consultations. Now there was a telephone interpreter service, which was far slower and meant no one was able to judge facial expressions: ‘It's a struggle speaking to a lot of people, especially in our practice, where we have a lot of non-English speakers’.

Mental health services had been stretched to their limit as a result of the pandemic, with waiting times of up to a year in Glasgow. By January 2021, some clients were ‘really struggling’ with things such as a sense of hopelessness and heightened levels of anxiety, increasing the demand and need for support services. A popular youth scheme was also not functioning. Those working in Glasgow expressed concerns about rising levels of domestic abuse and suicide issues:

[Now] the referrals coming through are…more round mental health -I know that was prominent right at the start but it’s kind of around social isolation; the groups are not face-to-face, and a lot of people are digitally excluded for one reason or another. So, a lot of people fleeing violence, relationships breaking down. *Links Worker*

As the pandemic progressed and the economy faltered, the strain on local statutory and non-statutory community services in Scotland had worsened. As one Links Worker explained, ‘The level of support that we’re giving [since Covid-19] is different because a lot of community organizations in Glasgow have just disappeared’. Referrals for food poverty and unemployment had also increased. Based in an area with a high number of asylum seekers, Ranesh, had made more referrals to food banks and ‘different community organizations that provide food for free for people’. Although these communities had already been high in need, ‘Since Covid, food poverty and [financial stress] has very much increased’.

Concerns were somewhat tempered by the fact that local communities were coming together to fill any voids and meet their local needs. One example given was that of an Arts and Music charity which was now providing a mental health help line for young people. Nevertheless, apprehension about future third-sector funding and contracts, and in particular the loss of smaller organizations, was a general concern for all stakeholders. With waiting lists and some clients opting to wait for services such as counselling to return to face-to-face, the fear was that there could be a substantial backlog of services once things begun to return to normal.

### Effects on Social Prescribing Coordinatorss

Managers from both the CLW scheme and SPRING emphasized that it was not just clients but workers who had been adversely affected by the pandemic. All SPCs said that they had found the period of working since the pandemic highly challenging, although for Link Workers supporting overstretched GP practices this was particularly acute. The added stress on their staff had not gone unnoticed by this Links Worker manager:

We talk a lot about the patient experience and patient wellbeing, but we need to keep in mind that the Links Workers are humans as well. They’ll have family issues that, they’ll be touched by Covid, they’ll have childcare issues…and we need to be mindful of that and be supportive. Because I think sometimes there can be too high an expectation on a Links Worker.

Some SPCs admitted that they had not found home-working easy: their work demanded a level of attention, confidentiality and privacy which could be hard to maintain in a shared household; ‘It’s making sure that I have my door closed and being aware of what my flat mates are doing as well…It's just hard’. To mitigate against isolation, SPCs in all schemes had regular on-line meetings with colleagues and line managers, which were considered as something of a lifeline in these difficult times.

## Discussion

Social constructionism studies how groups and individuals make claims to phenomena at different times and in various locations (Berger & Luckman, 1966), while intersectionality is a critical tool for understanding how socially constructed categories shape multiple dimensions of lived experience (Azhar & Gunn, 2021). Social interactions are based on tacit assumptions, which in times of crisis are disrupted. In the wake of Covid-19, participants in our study identified three particular concerns that relate to present and future social prescribing: identifying and supporting disadvantaged and vulnerable individuals and population groups, the move to digitalization, and the strains on statutory and third-sector services. We discuss these points in relation to the wider literature. Finally, we introduce our conceptualization of social prescribing as a collage, situated within a social texture in which intersecting inequalities are shaped by social challenges and disruptions. We will begin this section with a brief comparison between the three schemes as discussed by our study participants.

Overall, our findings reveal a complex, evolving social prescribing landscape in Scotland with ethos, management and delivery of services diverse and the project funding time limited and dependent on political will. While all stakeholders spoke of the need to promote individualized health solutions and reduce reliance on pharmaceutical medicine, representatives of schemes placed different emphasis on medical versus community models of care, and on different types of intersectionality. Stakeholders (medical and non-medical) in practice-attached schemes highlighted the importance of having Links Worker embedded within the practice, unlimited by referral criteria and working with people facing intersecting social, economic and ethnic barriers and disadvantages. SPRING representatives emphasized the advantages of locating social prescribing in community venues and ‘cutting out’ the medical association with social prescribing in the minds of clients. Working outside of NHS settings and serving isolated communities, CNs laid emphasis on technological education and connecting largely older clients with long-term health conditions to local or digital support services.

### Covid-19, Intersectionality and Digitalization

Intersectionality sheds light on the fact that individual and group inequities are shaped by interactions between multiple sites and levels of power (Kapilashrami & Hankivsky, 2018). It is not the pandemic itself directly that has challenged those in society, but the ‘engagement response’ which has exerted its profound effect on people’s lives in the form of government directives, uncertainties, fears, isolation and more (Bröer et al., 2021). Many factors can limit access to health care, those linked to socioeconomic disadvantage mentioned in this and other studies include: ethnicity and marginality, poor living conditions, low income, crowded or isolated living and disability (Carlisle, 2010). Those with complex needs who require the help of interprofessional teams already encounter barriers to engagement and, in times of social upheaval and heightened health risks, these disadvantages increase (Madden et al., 2020). While ethnicity is often presented as a barrier to health care, it is not ethnicity per se but a lack of prioritizing of the cultural and social needs of minority groups in decision-making that renders it a problem. This view is echoed in other studies, in which the higher observed incidence and severity of Covid-19 in minority groups has been associated with multiple socioeconomic, cultural and genetic factors, carrying on negative health trends that existed among minority communities prior to the pandemic (Khunti et al., 2020).

Initially caught up in ‘fire-fighting’ (such as delivering food and medicines), digital delivery soon became a priority for all three schemes. In practice, it took time and creativity on the part of schemes to put IT systems in place, a finding echoed in other reports on social prescribing services operational during Covid-19 (see, for example, Alliance for Healthier Communities (2021). By the end of the year the benefits and costs of digital communication had become more evident. While vital to the operation of health and allied services under social distancing measures, barriers to the use of technology noted in our own and other studies include digital literacy, access to technology, confidentiality and language have been noted in our own and other studies (Cárdenas et al., 2020; Fixsen et al., 2021). An important factor in the Covid-19 pandemic had been its unequal impact on different ethnic communities in Scotland (National Records of Public Health Scotland, 2020), and communications barriers can only have exacerbated this situation. Link Workers and GPs in our study described the existing telephone translation service as clumsy and impersonal. With digitalization of services set to continue, and access to health care already impaired among Scotland’s ethnic minorities (Meer et al., 2020), easier and faster access to health care through digital communication channels should be a priority.

### Impact on the Community/Third Sector

Prior to Covid-19, the voluntary and community (or third) sector in Scotland had experienced over a decade of reduced social funding (Mackenzie et al., 2020; Younan et al., 2020). With the advent of Covid-19, folding of third-sector schemes is predicted be more widespread (Lejac, 2021). A recent report on social prescribing in Scotland expressed concern about shrinking third-sector schemes at a time when non-medical services have never seemed more necessary (Lejac, 2021). Among schemes mentioned in our study, the folding of local youth services was regarded with particular concern at a time when young people have been deprived of social contact and many are struggling with their mental health (Youngminds, 2021). Helplines to domestic abuse victim services in the UK have also seen an increase in demand (Office for National Statistics, 2020), yet SPCs reported how a local abuse service in Glasgow which was a regular recipient of social prescribing referrals had closed its doors during Covid-19.

A new report commissioned by the ‘Royal Society of Edinburgh’s Post-Covid Futures Commission’ calls for decision-makers in Scotland to adopt a social prescribing approach to healthcare as a priority (Royal Society of Edinburgh, 2021a). This is partly in recognition of the potential that social prescribing has to prevent long-term conditions and lessen dependence on pharmaceutical drugs (Royal Society of Edinburgh, 2021b). Social prescribing is unsustainable without the substantial injection of funding into third-sector services that, especially in areas of high social deprivation and low income, cannot rely indefinitely on local good will and resourcing.

### The Chameleon Role of SPC

During Covid-19, the general adaptability of the SPC working practices placed them in a prime position to re-connect to service users remotely, signpost service users to functioning initiatives and create activities were none existed (such as one-to-one walking and online groups). Faced with high demand, but without the necessary services available, some SPCs had also been taking on a counselling and advocacy role for clients. While at times of upheaval an agile and flexible approach to problem management is often advantageous, the chameleon role of the SPC is also problematic. Social prescribing was never intended to be replacement for specialist agencies dealing with serious mental health issues, debt, housing, food poverty, domestic abuse, language difficulties or even counselling services, nor to subsidise service cuts due to public austerity (Dayson, 2017). Our study found evidence that some SPCs in deprived areas of Glasgow had felt impelled to help out in such cases in whatever ways were at their disposal. These factors may have altered the original ‘role description’ of the SPC/Links Worker as the links person connecting GP/referee and deliverer organization (Mercer et al., 2017; Polley, 2018), to something broader and more eclectic.

Changes in social and work conditions can have strong repercussions for those in helping professions such as social prescribing coordinators. Studies of previous pandemics suggest that health and social care workers have an increased risk of adverse mental health outcomes such as post-traumatic stress disorder and depression, and evidence is emerging of similar effects from Covid-19 (The Health Foundation, 2020). One study of lower paid health and social care staff in the Glasgow area indicated that these workers have higher rates of work-related health conditions than persons working in different sectors (NHS (Greater Glasgow and Clyde), 2012). SPCs are not strictly classified as working in health and social care; however, they largely work alone and now mostly at home. They also lack proper professional status, and some have insecure work contracts. All of these factors place an added strain on these workers already struggling with the complex caseloads they have received in the wake of the pandemic. In practice, partly due to the lack of professional status and limited career progression inherent in most SPC roles, some coordinators may not remain long in post. Two out of the three CNs we interviewed in April/May 2020 had left the scheme by January 2021 and were uncontactable. As recommendations for the future, we suggest a greater recognition of the agile and diverse role of the SPC. Full professionalization of the role however poses the danger of subsequent bureaucratization.

## Conclusions

Intersectionality sheds light on the fact that individual and group inequities are shaped by interactions between multiple sites and levels of power (Kapilashrami & Hankivsky, 2018). That services associated with social prescribing are considered as ‘peripheral’ suggests them to be undervalued in societies that reward medicalization and specialization more than inclusive community initiatives. The present arrangement of social prescribing services in the UK, including Scotland is, we argue, best conceived of as a collage of knowledges and practices, rather than a fully integrated model of care. We use the term ‘collage’ to suggest an assemblage superimposed upon a socio-political landscape that broadly encourages health inequities (Marmot, 2018; Mackenzie et al., 2020; Gibson et al., 2021). Those most in need of social prescribing services are less likely to break through disadvantage while they are situated within a social texture in which inequities in education, health, social and living arrangements intersect with one another. As exemplified by the pandemic, inequalities are intersecting and are shaped by social challenges and disruptions, often for the worse rather than the better. At the same time, a shakeup of the normal order can provide a means of better identifying these intersections and specific areas requiring improvement. The pandemic has demonstrated that preparedness for health crises lies not only in supporting a robust mainstream health service at all times, but also the auxiliary arms so vital to its sustainability and for halting the further escalation of health and social inequalities. It is imperative that governments sponsor initiatives such as social prescribing less to score political points and to shift responsibility from primary healthcare to the individual and community, and more as an adjunct to robust policies that promote social, educational and economic equity, including for minority groups. Right now, this requires the necessary injections of funds and resources into communities that continue to suffer through crises.

### Limitations and Strengths of Study

Limitations of this study include the failure to speak with patients due to Covid-19 restrictions and the relatively small sample of stakeholders representing schemes who were contacted for the first interview and available for the second interview. However, we have been able to consider social prescribing in diverse settings within one country by speaking with various stakeholders of three different types of social prescribing schemes. This gave us a bird’s eye view of social prescribing in Scotland and from this we developed a high-level understanding of its benefits and challenges. Moreover, we interviewed participants during the Covid-19 pandemic and lockdown and were able to record in real-time how schemes adapted to the ongoing challenges. Critical sociological studies should, we argue, continue to address uncomfortable issues such as highlighting the politically unstable and asymmetrical socioeconomic landscape in which individually well-designed health and social schemes operate and the limitations this imposes upon them.

## Supplemental Material

sj-pdf-1-qhr-10.1177_10497323211064229 – Supplemental Material for Supporting Vulnerable Populations During the Pandemic: Stakeholders’ Experiences and Perceptions of Social Prescribing in Scotland During Covid-19 Click here for additional data file. Supplemental Material, sj-pdf-1-qhr-10.1177_10497323211064229 for Supporting Vulnerable Populations During the Pandemic: Stakeholders’ Experiences and Perceptions of Social Prescribing in Scotland During Covid-19 by Dr Alison Fixsen, Dr Simon Barrett and Michal Shimonovich in Qualitative Health Research

## Notes

1 Salutogenic refers to an approach that focuses on multiple factors that support human health and wellbeing, rather than solely on pathogenic factors.

2 The University of the Highlands and Islands are currently carrying out a mixed method evaluation of social prescribing in rural and remote Scotland (see: https://pure.uhi.ac.uk/en/projects/social-prescribing-for-remote-and-rural-scotland-a-mixed-methods-)
