# Supplementary material for: Supporting Vulnerable Populations During the Pandemic: Stakeholders’ Experiences and Perceptions of Social Prescribing in Scotland During Covid-19
Source: Qual Health Res. 2021 Dec 30;32(4):670–82. doi: 10.1177/10497323211064229 (PMC8948336; doi:10.1177/10497323211064229)
Supplement: sj-pdf-1-qhr-10.1177_10497323211064229 – Supplemental Material for Supporting Vulnerable Populations During the Pandemic: Stakeholders’ Experiences and Perceptions of Social Prescribing in Scotland During Covid-19 [file sj-pdf-1-qhr-10.1177_10497323211064229.pdf]

Questions for individual interviews with professionals

1. Can you tell me about your role with the Social Prescribing Service?
2. Could you describe how the service operates from your perspective?
   - Referral system and referral criteria
   - Support offered to service users
   - How service users 'exit' the service

3. In what ways has communication and partnerships between the Social Prescribing Service and the voluntary and community sector been established?

4. What do you see are the key strengths of the social prescribing model?

5. What have been the main challenges in delivering the service?

6. From your experience, in what ways at all do existing social prescribing services support marginalised members of communities (such as homeless, substance abusers, people on probation) in Glasgow?

7. What differences if any do you think the service could make to such people?

8. To what extent has the voluntary and community sector succeeded in providing support for marginalised members of communities in Glasgow?

9. Are there any recommendations you would offer for improving access to marginalised members of communities?

Table 1. Summary of three social prescribing schemes across Scotland: Links worker programme, SPRING and mPower

<!--Col Count:4-->	Links Worker Programme	SPRING	mPower
Description	Started as a pilot in 2014 as a way to support GPs in practices in Glasgow's most deprived areas. Currently 34 link workers are attached to practices in Glasgow	SCHW in Scotland and HCLA in Northern Ireland teamed up to fund social prescribing schemes in 30 rural and urban areas, including Glasgow.	The mPower project supports social prescribing and eHealth interventions across borders in the West of Scotland.
Theoretical framework	Part of Deep End strategy to reduce health inequalities and reverse	Theory of change model[b]	Personalized well-being plans, supporting self-

<!--Col Count:4-->	Links Worker Programme	SPRING	mPower
	Inverse care law[a]		management[c]
Scheme location for this study	Glasgow Deep End general practice surgeries.	Community centres in Glasgow	Isles of Barra, N. and S Ulst, Benbecula, Harris and Lewis.
Estimated Scheme duration	Oct 2014 –	April 2018-April 2023	Late 2017-Dec 2021
Funders	Scottish Government	The National Lottery Fund	European Union's INTERREG VA Programme, managed by the Special EU Programmes Body (SEUPB). Match funded by Scottish Government.
Management organisations	Health and Social Care Alliance	Scottish Communities for Health and Wellbeing (SCHW) in Scotland	NHS Western Isles employ the social prescribers. The SCVO manages communications with third sector.
Financial investment	The Scottish Government paid each participating practice between around £50,000. Up to £17,500 is spent on adapting the Links Worker Programme into the surgery.	The National Lottery Fund provides £3 million in support.	The project budget is €8,700,000 (for all three regions)
Social prescriber location	Based in general practice surgeries	Based at voluntary and community organisations	Serve rural communities and based in regions.
Designated name of SPC	Community Links Practitioner	Social Prescribing Advisors	Community Navigator
Patient criteria	All patients over 18 registered with the general practice, including those with complex needs. Patients can be referred or self-	Adults over 18 who have been referred. Focus on patients with low level needs, e.g. social isolation, loneliness, physical activity.	Adults over 18, but predominantly over 65s with long term conditions.

<!--Col Count:4-->	Links Worker Programme	SPRING	mPower
	refer.		

[a]GPs at the Deep End. Retrieved 28 August 2021, from: https://www.gla.ac.uk/media/Media_513883_smxx.pdf

[b]SPRING Social Prescribing Project Evaluation Report 2019-20. Retrieved 28 August 2021, from: https://www.tnlcommunityfund.org.uk/media/insights/documents/SPRING_EvaluationReport.pdf?mtime=20210618134201&focal=none

[c]About mPower. Retrieved 28 August 2021, from: https://mpowerhealth.eu/about

<!--Col Count:3-->Interview Date	Role	Programme
01/04/2020<br>18/02/2021	Manager	SPRING
05/04/2020	GP	Deep End Practice
07/04/2020<br>02/02/2021	Community link worker (CLW)	Community Link Worker Scheme
09/04/2020	Community link worker (CLW)	Community Link Worker Scheme
20/04/2020<br>12/02/2020	Social prescribing advisor	SPRING
21/04/2020<br>03/02/2021	GP	Deep End Practice
28/04/2020<br>27/01/2021	Manager	Community Link Worker Scheme
28/04/2020	Manager	Third sector organisation
28/04/2020	Researcher	Scottish Government
01/05/2020	GP	Deep End Practice
05/05/2020	GP	Deep End Practice
07/05/2020	Community volunteer	Third sector organisation
11/05/2020	GP	Deep End Practice
14/05/2020<br>04/02/2021	Community link worker (CLW)	Community Link Worker Scheme
29/05/2020	Community volunteer	Third sector organisation
19/05/2020	Researcher	University
24/05/2020	Manager	mPOWER
26/05/2020	Manager	mPOWER
04/06/2020	Community navigator	mPOWER
11/06/2020<br>05/02/2021	Community navigator	mPOWER
16/06/2020	Community navigator	mPOWER
18/06/2020	Researcher	University

Key: Table of participants with dates of interviews and role description. All names are pseudonyms.
